# Ectonucleoside triphosphate diphosphohydrolases and ecto-5′-nucleotidase in purinergic signaling: how the field developed and where we are now

**DOI:** 10.1007/s11302-020-09755-6

**Published:** 2020-12-17

**Authors:** Herbert Zimmermann

**Affiliations:** Goethe University, Institute of Cell Biology and Neuroscience, Max-von-Laue-Str. 13, 60438 Frankfurt am Main, Germany

**Keywords:** Adenosine, ATP, Ecto-5′-nucleotidase, E-NTPDase, Geoffrey Burnstock, History

## Abstract

Geoffrey Burnstock will be remembered as the scientist who set up an entirely new field of intercellular communication, signaling via nucleotides. The signaling cascades involved in purinergic signaling include intracellular storage of nucleotides, nucleotide release, extracellular hydrolysis, and the effect of the released compounds or their hydrolysis products on target tissues via specific receptor systems. In this context ectonucleotidases play several roles. They inactivate released and physiologically active nucleotides, produce physiologically active hydrolysis products, and facilitate nucleoside recycling. This review briefly highlights the development of our knowledge of two types of enzymes involved in extracellular nucleotide hydrolysis and thus purinergic signaling, the ectonucleoside triphosphate diphosphohydrolases, and ecto-5′-nucleotidase.

## Introduction

Ectonucleoside triphosphate diphosphohydrolases (E-NTPDases) and ecto-5′-nucleotidase (eN) are only part of a broader spectrum of extracellular nucleotide-metabolizing enzymes, including ectonucleotide pyrophosphatase/phosphodiesterases, alkaline phosphatases, prostatic acid phosphatase, or extracellular ATP-regenerating enzymes [1, 2]. Yet, E-NTPDases and eN have been the enzyme axis most extensively studied regarding purinergic signaling. Geoff Burnstock maintained great interest in the mechanisms of extracellular nucleotide breakdown as these control purinergic receptor activity. This brief review is dedicated to Geoffrey Burnstock (1929–2020) as the leading scientist and promotor in the field, founder and chief editor of this journal, wonderful colleague, and friend.

## Nucleoside triphosphate diphosphohydrolases

### Early studies

**Fig. 1:**
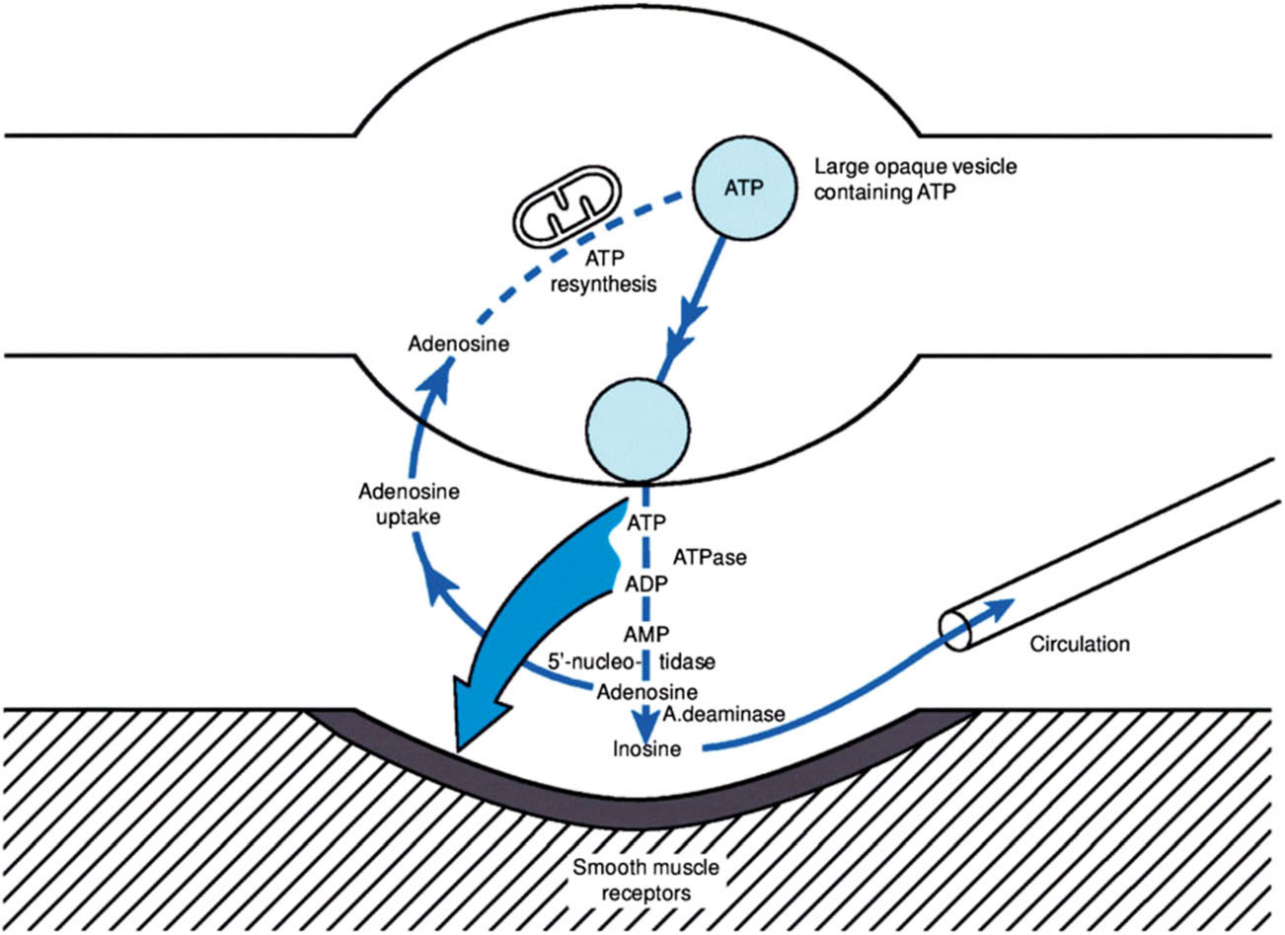
Schematic representation of synthesis, storage, release, and inactivation of ATP at purinergic nerves as depicted by Burnstock for purinergic neuromuscular junctions in 1972 [3]. Reproduced with permission from the American Society for Pharmacology and Experimental Therapeutics

Regarding the fate of ATP released from nerve endings, Geoff Burnstock, in his seminal review of 1972 [3], discards the possibility that it can directly be recycled. He strongly supports the notion that it is broken down by extracellularly located enzymes via ADP and AMP to adenosine, which is then recycled into the nerve ending for intracellular resynthesis of ATP. He develops a model of synthesis, storage, release, and inactivation of ATP at the purinergic neuromuscular junction that still holds today (Fig. 1).

Evidence for extracellular hydrolysis of ATP in tissue perfusates was provided already in the 1930s. But biochemical approaches to a mechanistic analysis were developed later. Since ATP is hydrolyzed intracellularly and by broken tissue, convincing evidence for cell surface-located ATP hydrolysis could initially only be obtained by analysis of dispersed and intact cells. First evidence was provided in 1945 in carefully washed bull spermatozoa by T. Mann in Cambridge [4]. More detailed reports followed this pioneering study [5, 6]. When analyzing nucleated avian erythrocytes, Wladimir A. Engelhardt and Tatjana Wenkstern realized that not only ATP but also ADP or ITP were hydrolyzed [7, 8]. Catalytic activity had an alkaline pH optimum and was blocked by EDTA (ethylenediaminetetraacetic acid). These two authors introduced the term *ecto-ATPase* in 1955 [9] as well as the terms *ectoenzyme* and *ecto-apyrase* (Engelhardt, 1958, held at the International Symposium on Enzyme Chemistry, Tokyo and Kyoto, 1957, [7] and Wenkstern and Engelhardt in 1959) [8].

### Objections and a solution

Yet, the function of this “ectoenzyme” remained obscure. Nucleotides appeared not to be present in appreciable amounts in the extracellular medium. ATPase activity was solely known to relate to cellular energetics and cellular metabolism. Could it have something to do with active transport of substances across the plasma membrane or the control of cell permeability? [7]. This problem persisted for a very long time. Biochemists would not agree that an energy-rich substance such as ATP would at all be released from cells under physiological conditions. And if so, the free enthalpy of hydrolysis had to be employed somehow for an energy-driven cellular process. It would not simply evaporate. This is the merit of Geoff Burnstock: extracellular nucleotide hydrolysis makes sense in the light of purinergic signaling.

### Biochemical analysis

In spite of these uncertainties, an increasing number of studies using various cellular systems analyzed the catalytic properties of extracellular nucleotide hydrolysis. The results varied to some extent between individual studies. In retrospect, this is not at all surprising since several “ATPases” exist in the plasma membrane and, even more, several ectoenzymes capable of hydrolyzing extracellular ATP may coexist in the same tissue. But some consensus was achieved that the “enzyme” is a glycosylated membrane integral protein, that the underlying catalytic activity is activated by Ca^2+^ or Mg^2+^ in the millimolar range and inhibited by EDTA, and that catalytic activity is highly sensitive to SH reagents but insensitive to inhibitors at concentrations which inhibit mitochondrial ATPase and Na^+^/K^+^-ATPase. *K*_m_ values for ATP were in the low millimolar range. Both purine and pyrimidine nucleotides were hydrolyzed, albeit with differing efficiency. In the 1980s, first attempts were made to purify the ectoenzyme(s). The high detergent sensitivity turned out to be a major obstacle for enzyme purification because the monomeric forms retain little catalytic activity. These early studies were summarized in several reviews [10–20].

### Purification and molecular cloning

**Fig. 2:**
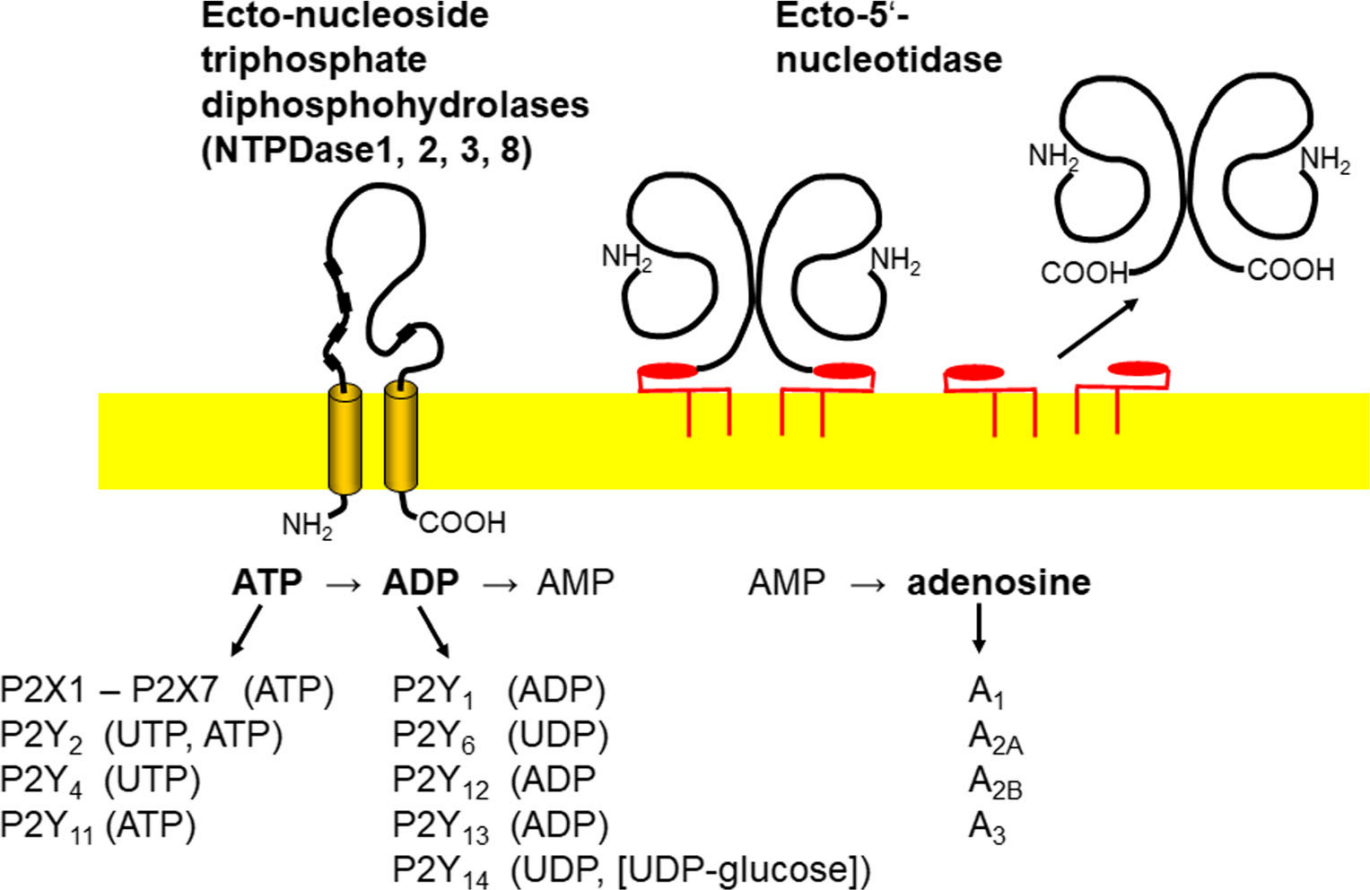
Membrane topology of NTPDases 1, 2, 3, and 8 and eN (ecto-5′-nucleotidase). The boxes in the NTPDase extracellular loop represent the position of the apyrase conserved regions. eN is GPI-anchored. The GPI anchor may be cleaved by endogenous phospholipases resulting in the release of the enzyme into the interstitial space. NTPDases have the potential to form homo-oligomeric complexes (dimers to tetramers). eN exists and functions as a noncovalent dimer [1]. The hydrolysis cascade is shown for ATP to adenosine. But it applies also to other nucleoside triphosphates (NTP → NDP → NMP; NMP → nucleoside). Purinergic receptors activated by nucleotides and adenosine are indicated below

The rise of molecular genetics made all the difference. Sequence information from purified proteins made it possible to identify the encoding cDNA, followed by heterologous expression and analysis of the protein. Moreover, sequence comparison allowed the identification of paralogues and of orthologues in other species. This was achieved by converging efforts of several laboratories. An ATP diphosphohydrolase was first purified to homogeneity by Christoforidis et al. in 1995 [21] from human placenta. It turned out that the peptide sequences obtained corresponded to a functionally as yet unidentified lymphoid cell activation protein (Cluster of differentiation 39, CD39) that had been cloned and sequenced shortly before [22]. Of note, this was not known to Christoforidis et al. when they submitted their paper. Moreover, a soluble apyrase was cloned from potato tubers in 1996 which was found to be related to CD39 and known apyrases from other organisms. Apparently, there was a group of widely conserved enzymes whose sequences shared typical features such as the “apyrase conserved regions” [23]. In the same year, this laboratory demonstrated ecto-apyrase activity of CD39 by expression in COS-7 cells [24]. Moreover, peptide sequences from a bovine aorta-derived ATP diphosphohydrolase revealed identity with CD39 [25]. Similarly, expression of CD39 in COS-1 cells confirmed its ecto-ADPase activity and highlighted its role as a prime endothelial thromboregulator [26]. The ice was broken. While it was originally thought that there was only a single mammalian “ecto-apyrase,” a paralog was soon sequenced and expressed by Kegel et al., in 1997 [27]. Surprisingly, it turned out to preferentially hydrolyze ATP and appeared to function as an “ecto-ATPase” rather than an “ecto-apyrase” [28]. Moreover, four paralogs were identified in 1998 in the human genome, demonstrating that an entire gene and protein family must exist [29]. We now know that eight paralogs are encoded in the mammalian genome, all hydrolyzing nucleotides only, four of which are typical surface-located ectonucleotidases (NTPDase1, 2, 3, and 8) (Fig. 2). Related enzymes are found in invertebrates, plants, yeast, protozoans, and bacteria [30]. E-NTPDases share common sequence motifs with members of the ASKHA (acetate and sugar kinases/Hsc70/actin) superfamily of phosphotransferases [1, 31, 32] .

The years following envisaged impressive progress in further characterizing proteins and genes, using mutation studies, developing inhibitors, resolving atomic structures, and analyzing their function in physiological and pathophysiological conditions. The four surface-located E-NTPDases are glycosylated and share their general membrane topology with two transmembrane domains, which play an important role in function and regulation of the enzymes in addition to anchoring the proteins in the plasma membrane (Fig. 2). The formation of oligomers is essential for full catalytic activity. The biochemical properties of the E-NTPDases, their splice variants, and their tissue distribution have been reviewed in detail [1, 19, 20, 32–36].

### Confusing nomenclature

Considerable confusion existed regarding nomenclature. Different names had been assigned by different groups and to individual paralogues. Moreover, the often-used term ecto-ATPase for the ecto-ATP diphosphohydrolase appeared misleading since it disguised the fact that also ADP (an agonist of several P2Y receptors) is hydrolyzed with AMP as the final hydrolysis product. Moreover, not only ATP and ADP but also other nucleoside tri- and diphosphates were hydrolyzed. The author of this article thus put together a nomenclature committee which finally met at the conference on “Ecto-ATPases and related ectonucleotidases” held in Diepenbeek, Belgium, in 1999 where it was agreed to apply a strictly biochemical enzyme nomenclature and to name this new protein family ectonucleoside triphosphate diphosphohydrolase family (E-NTPDase family) (EC: EC 3.6.1.5) and its individual members NTPDase1, NTPDase2, and so on [35, 37]. While the name CD39 is frequently used for NTPDase1 in studies merely relating to its catalytic function, the author holds that the enzyme nomenclature should be applied.

### Crystal structures and catalytic cycle

**Fig. 3:**
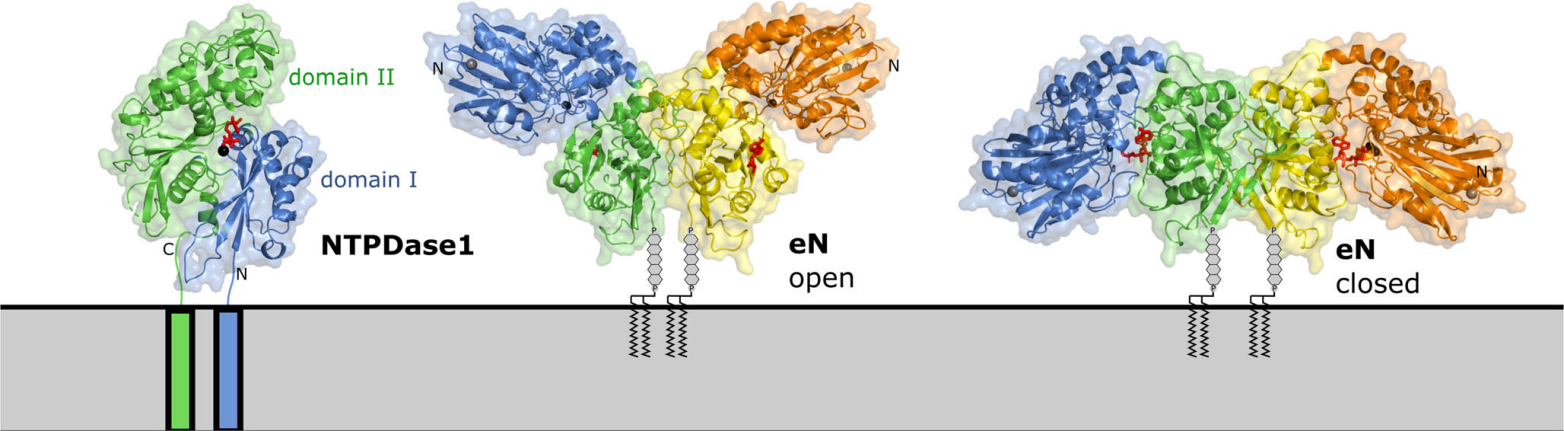
Ectodomain structure of NTPDase1 and eN. To mark the active site of rat NTPDase1 (chain A of protein data bank [pdb] id 3zx3), the non-hydrolysable ATP analogue AMPPNP (β,γ-imidoadenosine 5′-triphosphate) (red) and a calcium ion (black sphere) have been superimposed from rat NTPDase2 structure (pdb id 3cja). For the homodimeric eN, the domains of one monomer are depicted in blue and green, whereas the other subunit is shown in yellow and orange. The catalytic zinc ions are shown in black and the structural Ca^2+^ ions in gray. Adenosine (red) is bound to the C-terminal domains of the open state structure of eN (pdb id 4h2i), and AMPCP (adenosine 5′-[α,β-methylene]diphosphate) (red) is bound to the active site in the closed state structure (pdb id 4h2i). The figure was kindly provided by Norbert Sträter, Leipzig, Germany

Of central importance for understanding the molecular mechanisms of hydrolysis and the development of inhibitors was the resolution of crystal structures of E-NTPDases. First structures were obtained of the extracellular domain of rat NTPDase2 [38] and a related soluble NTPDase of the pathogenic bacterium *Legionella pneumophila* (*Lp*NTPDase1), which is secreted into the replication vacuole [39]. The crystal structures revealed a pseudo-symmetrical arrangement of two extended RNase H fold repeats that is also found in other members of the actin structural superfamily. Two structural domains are formed which are characterized by a central mixed β-sheet and a peripheral layer of mainly α-helices. Co-crystals with substrate analogs allowed to identify the catalytic site and to propose a catalytic mechanism involving the individual apyrase conserved regions. The same catalytic site is employed in the hydrolysis of nucleoside di- and triphosphates. During the catalytic cycle, the domains undergo rotational movements supporting the idea that the previously described impact of transmembrane helix dynamics on activity is achieved by coupling to a domain motion (Fig. 3) [40, 41].

### Development of inhibitors

Multiple studies have highlighted the involvement of ectonucleotidases in pathological conditions. The interplay of ectonucleotidases with the nucleotide and adenosine receptor systems has come increasingly into focus. Alterations in extracellular nucleotide and adenosine levels can increase or decrease P2 receptor and P1 receptor activity. The development of potent and subtype-specific ectonucleotidase inhibitors thus appeared mandatory [42]. This was a field Geoff Burnstock was particularly interested in. In the 1990s, his group published a series of papers on “ecto-ATPases,” which mostly focused on the characterization of enzyme inhibitors [13]. The development of potent and specific inhibitors turned out to be a challenge. Inhibitors should not affect nucleotide receptors or other types of ectonucleotidases—which all share nucleotide-binding sites. And they should not become hydrolyzed. While several E-NTPDase inhibitors have been developed, potent subtype-specific inhibitors are scarce. Most of these are ATP analogs. Other classes concern polyoxometalates, negatively charged metal complexes, anthraquinone derivatives, Schiff bases of tryptamine, quinoline derivatives, and thiadiazolopyrimidones [43–46]. The elucidation of the molecular structure of mammalian E-NTPDases now permits a structure-guided approach of inhibitor development with the ultimate goal of drug design [47].

### Therapeutic approaches

Equally important were studies which generated subtype-specific antibodies for analyzing the distribution of the individual enzymes in mammalian tissues [42] and the generation of mice in which individual NTPDases were deleted. The first gene encoding a mammalian NTPDase deleted from the germline was *Entpd1* [48]. The study proved its fundamental role in hemostasis and thrombosis. This was followed by the deletion of *Entpd2*, the gene encoding NTPDase2, which allowed to analyze the function of the enzyme in taste buds [49], followed by the deletion of NTPDas3 [50]. Moreover, transgenic overexpression of NTPDase1 in mice or pigs permitted insight into its role in multiple organ systems. One outcome was the attenuation of myocardial infarction by decreasing infarct size [51–53], confirming previous results emphasizing the important role of ATP hydrolysis and in particular of NTPDase1 in the interplay with nucleotide receptors in the control of vascular function [54, 55]. Moreover, the benefit of administration of soluble apyrase or of induction of NTPDase1 by adenoviral vectors on several models of organ transplantation has been investigated [56]. By now, multiple organ systems and disease models including cancer, immunosuppression, and inflammation have been studied. Recently, the clinical evaluation of anti-NTPDase1 monoclonal antibodies for cancer therapy has been initiated [57]. Only a selection of overviews can be cited here [58–70].

## Ecto-5′-nucleotidase

### Biochemical properties

This enzyme was first described in extracts of heart tissue by J.L. Reis in 1934 who named it “5-nucleotidase” [71]. He realized that “5-nucleotidase” differs from nonspecific phosphatases already known at the time as it showed high specificity towards nucleoside monophosphates (Fig. 2). In contrast to NTPDases, 5′-nucleotidase was intensively investigated early on [72, 73]. In 1974, it was shown that 5′-nucleotidase is an ectoenzyme in several cell types [74–76]. As a result of adenosine production, scavenging of extracellular nucleotides (including nutrition), involvement in vasodilation, neurotransmission, or hemostasis had been described [77]. The glycoprotein eN is a major enzyme producing adenosine from extracellular AMP and thus for activation of adenosine receptors [78]. Before this context had been elucidated, eN was widely used as a membrane marker and for the analysis of plasma membrane recycling [79]. Eukaryotic eN functions as a noncovalent dimeric Zn^2+^-binding protein, with reported *K*_m_ values for AMP between 1 and 50 μM. ATP and ADP are competitive inhibitors of mammalian eN with *K*_i_ values in the low micromolar range. This is important, since due to feed forward inhibition, adenosine formation from ATP or ADP will be considerably delayed until extracellular nucleotide levels have fallen into the micromolar range [1].

While it was originally assumed that eN is an integral membrane protein, it was shown by several groups that it can be released by phosphatidylinositol-specific phospholipase C and thus must be anchored to the plasma membrane by a glycosylphosphatidylinositol (GPI) anchor [80]. Primary structures were first obtained for the enzyme from rat liver [81], human placenta [82], and the brain of the electric ray [83]. Sequence comparison revealed that eN can be grouped into the calcineurin superfamily of dinuclear metallophosphatases with multiple members in prokaryotes, invertebrates, and vertebrates. The molecular and functional properties of eN have been reviewed [1, 84, 85]. Interestingly, humans express several transcript variants [86].

### Nomenclature

As for NTPDases, the nomenclature of 5′-nucleotidases was initially confusing. Apparently, there existed also soluble forms. Whereas some shared properties with eN, others clearly differed regarding catalytic properties. Therefore, an attempt was made by the author of this article to classify the various types of 5′-nucleotidases, and a new nomenclature was suggested [80]. One of the soluble forms was assigned to eN, generated by cleavage of the GPI anchor. At that time, no sequence information was available for soluble 5′-nucleotidases. After the sequences of the six intracellular and soluble 5′-nucleotidases had been revealed, the nomenclature was adapted accordingly [87]. CD73 (cluster of differentiation 73) is frequently used as an alternative name in studies addressing eN.

### Crystal structures and inhibitors

Crystal structures were first obtained for *Escherichia coli* 5′-nucleotidase which served as a model for mammalian eN [88, 89]. In 2012, crystal structures of both the open and closed form of human eN lacking the membrane anchor were determined [90, 91]. These studies revealed an extensive active site closure movement involving the N- and C-terminal domains of the eN monomer, which is thought to be necessary for human eN catalysis, permitting substrate binding and product release (Fig. 3). In addition, the active site closure movement may control eN substrate specificity towards AMP and thereby inhibition by ADP and ATP. It is now possible to design structure-based potent and selective small molecule inhibitors for future drug development. This is important as the hydrolysis product adenosine is involved in numerous pathologies. Progress had been made with several naturally occurring phenolic compounds and flavonoids or anthraquinone dye derivatives [44, 92]. A catalytically active soluble rat eN purified after heterologous expression in insect cells [93] has been widely used for drug screening. Recently, small molecule inhibitors with subnanomolar *K*_i_ values at human and rat eN could be developed, which are derivatives of purine and pyrimidine nucleotides. Moreover, high-resolution co-crystal structures revealed insight into the binding mode and represent an excellent basis for drug development [57, 94–96]. Similarly, monoclonal antibodies are applied as inhibitors of eN and may be employed as therapeutic agents [97–99].

### Highly relevant for adenosine signaling

Ecto-5′-nucleotidase plays an important role in tissue homeostasis and pathology in many organ systems and in acute and chronic inflammation [2]. This is particularly relevant in the context of acute and chronic types of injury, where eN is essential for maintaining tissue integrity and recovery [69, 86]. Important insight was obtained by targeted disruption of the *Nt5e* gene in mice revealing that vascular leakage was significantly increased in multiple organs and identifying the enzyme as a critical mediator of vascular leakage in vivo [100]. Moreover, genetic deletion of eN in mice is associated with a proinflammatory phenotype suggesting that eN-mediated adenosine formation represents a key innate mechanism to attenuate tissue inflammation [101, 102]. Behavioral analyses of eN knockout mice suggest that eN is involved in the regulation of learning and memory and psychomotor coordination [103]. Numerous studies analyzing *Nt5e*-depleted mice followed [85]. More recently, eN has gained considerable attention as a target for cancer treatment. Both ATP and adenosine accumulate at high levels in inflammatory and tumor sites. They play a central role in immune cell regulation and tumor cell proliferation. eN is upregulated in various types of cancer. The immunosuppressive adenosine impairs antitumor responses and enhances tumor growth and metastasis. Targeted eN (as well as NTPDase1) therapy using inhibitors is therefore an important approach to effectively control tumor growth [57, 99, 104–106].

### Résumé

Fifty years after establishing the concept of purinergic signaling by Geoff Burnstock and after about 80 years following the discovery of the two types of ectonucleotidases and numerous studies which elucidated their functional and structural properties, the time is now ripe for harvest. This concerns in particular the application of new tools for identifying the pathophysiological involvement of the enzymes in purinergic signaling in the various organ systems and the development of tailored therapies for human diseases.

## Data Availability

Not applicable.
